# Irisin Protects against Loss of Trabecular Bone Mass and Strength in Adult Ovariectomized Mice by Stimulating Osteoblast Activity

**DOI:** 10.3390/ijms24129896

**Published:** 2023-06-08

**Authors:** Giuseppina Storlino, Manuela Dicarlo, Roberta Zerlotin, Patrizia Pignataro, Lorenzo Sanesi, Clelia Suriano, Angela Oranger, Giorgio Mori, Giovanni Passeri, Silvia Colucci, Maria Grano, Graziana Colaianni

**Affiliations:** 1 Department of Clinical and Experimental Medicine, University of Foggia, 71100 Foggia, Italy; 2 Department of Precision and Regenerative Medicine and Ionian Area, University of Bari, 70124 Bari, Italy; manuela.dicarlo@uniba.it (M.D.); maria.grano@uniba.it (M.G.); 3 Department of Translational Biomedicine and Neuroscience, University of Bari, 70124 Bari, Italy; 4 Unit of Clinica e Terapia Medica, Department of Medicine and Surgery, University of Parma, 43121 Parma, Italy

**Keywords:** Ovx-mice, irisin, bone loss, estrogen, subchondral bone

## Abstract

Irisin is a peptide secreted by skeletal muscle that plays a major role in bone metabolism. Experiments in mouse models have shown that administration of recombinant irisin prevents disuse-induced bone loss. In this study, we aimed to evaluate the effects of irisin treatment for the prevention of bone loss in the ovariectomized (Ovx) mouse, the animal model commonly used to investigate osteoporosis caused by estrogen deficiency. Micro-Ct analysis conducted on Sham mice (Sham-veh) and Ovx mice treated with vehicle (Ovx-veh) or recombinant irisin (Ovx-irisn) showed bone volume fraction (BV/TV) decreases in femurs (Ovx-veh 1.39± 0.71 vs. Sham-veh 2.84 ± 1.23; *p* = 0.02) and tibia at both proximal condyles (Ovx-veh 1.97 ± 0.68 vs. Sham-veh 3.48 ± 1.26; *p* = 0.03) and the subchondral plate (Ovx-veh 6.33 ± 0.36 vs. Sham-veh 8.18 ± 0.41; *p* = 0.01), which were prevented by treatment with a weekly dose of irisin for 4 weeks. Moreover, histological analysis of trabecular bone showed that irisin increased the number of active osteoblasts per bone perimeter (Ovx-irisin 32.3 ± 3.9 vs. Ovx-veh 23.5 ± 3.6; *p* = 0.01), while decreasing osteoclasts (Ovx-irisin 7.6 ± 2.4 vs. Ovx-veh 12.9 ± 3.04; *p* = 0.05). The possible mechanism by which irisin enhances osteoblast activity in Ovx mice is upregulation of the transcription factor *Atf4*, one of the key markers of osteoblast differentiation, and osteoprotegerin, thereby inhibiting osteoclast formation.

## 1. Introduction

Postmenopausal osteoporosis is characterized by a reduction in bone mass and altered bone microarchitecture, leading to increased bone fragility and risk of fractures [1]. Estrogen deficiency after ovariectomy in mice and menopause in women is associated with significant bone loss, itself characterized by malfunctioning of the modeling and remodeling cycle. This phenomenon is due to an increase in resorption processes by osteoclasts, without a balance from deposition processes, thus leading to bone loss [2,3]. In addition, in vivo experiments conducted in Ovx mouse and guinea pig models showed increased osteoarthritic processes and negative impact, particularly on the subchondral bone, caused by estrogen deficiency [4,5]. Likewise, the skeletal muscles in postmenopausal women are affected by the loss of ovarian hormone production, thus accelerating the decline in muscle strength [6].

It is well known that muscle tissue and bone tissue are closely associated and connected both anatomically and through a molecular network of muscle-derived myokines with hormone-like functions influencing bone. One of these myokines is irisin, a molecule produced by cleavage of its precursor, fibronectin type III domain-containing 5 (Fndc5) [7], which exerts an anabolic effect on bone [8]. In vitro studies have shown that irisin treatment increases osteoblast differentiation [9], inhibits osteocyte apoptosis [10,11], and indirectly affects osteoclastogenesis by increasing the expression of osteoprotegerin (OPG) [12]. However, the direct effect on osteoclasts remains to be elucidated. Estell et al. demonstrated that irisin promotes osteoclastogenesis [13], while another study performed by Zhang et al. showed the inhibitory effect of irisin on osteoclast formation [14]. These opposite results were probably due to the irisin treatment protocol, which was different between the two studies; chronic treatment (2 months) with irisin increased osteoclastogenesis [13], while its administration for a shorter time course (7 days) inhibited it [14]. Furthermore, other authors have shown that irisin promotes proliferation but inhibits differentiation of osteoclast precursors [15].

In vivo, irisin has been shown to increase cortical bone mass in healthy young mice [16] to prevent disuse-induced trabecular bone loss and to promote osteogenesis and osteocyte viability, while inhibiting bone cell senescence in osteo-sarcopenic mice [10,17,18]. As evidence of the key role of irisin in bone formation, Zhu et al. showed that a knockout mouse for *Fndc5* had a lower bone density and significantly delayed bone mineralization compared with normal mice [19]. Recent studies have also shown that irisin treatment accelerates fracture-healing processes in mice by modulating the expression of angiogenic, inflammatory, and osteogenic factors [20,21,22].

Since its discovery, the effect of irisin on bone loss due to estrogen deficiency has been less explored. One study observed that chronic exercise increased the expression of the irisin precursor, *Fndc5*, in the gastrocnemius and soleus muscles of ovx mice. Regression analysis showed that *Fndc5* mRNA levels correlated positively with trabecular BMD in the femurs and tibia of ovx mice [23]. Another study found that exercise-related improvement in bone mass in 3 month old ovx mice was attenuated by blocking irisin receptor signals using biweekly injection of cyclo RGDyk protein, an αV inhibitor/antagonist [24]. However, a limitation of this study was that cyclo RGDyk is not a selective inhibitor for the irisin receptor αV/β5, as it also blocks the αvβ3 integrin signaling pathway [25], thus possibly directly modulating the skeletal response to mechanical loading. Only one study analyzed the effect of exogenous administration of recombinant irisin in 10-week-old mice, showing that irisin-treated ovx mice had higher trabecular bone mass and mechanical strength than vehicle-treated ovx mice. However, in this study, ovariectomy was performed on mice that were still young and had not yet reached peak bone mass. Presumably, this explains why no significant bone loss caused by estrogen removal was observed when comparing ovx-vehicle mice with sham-operated mice [26].

In humans, clinical studies found that irisin serum levels were lower in postmenopausal women [27], particularly in those with osteoporotic fractures [28,29], and inversely correlated with vertebral fragility fractures [30]. Further studies demonstrated that low irisin levels in postmenopausal women were associated with an increased risk of hip fractures, suggesting that measuring myokine levels could provide predictive information as a useful index to identify postmenopausal women with higher risk of fracture [31].

Treatment with 100 μg/kg of recombinant irisin administered intermittently (once a week) for 28 days has been shown to be effective in preventing bone loss in other mouse models of osteoporosis [10,17,18]. Considering the evidence in human studies and the relevance of a possible irisin-based therapy to prevent bone loss caused by estrogen decline, our study aimed to determine the effects of irisin on bone loss prevention in adult ovx mice treated with irisin, following the intermittent protocol. At the end of the treatment, we performed a quantitative assessment of the bone microarchitecture of both the cortical and trabecular bone, using 3D imaging techniques such as computed microtomography (micro-CT) and three-point bending tests to assess the biomechanical properties of long bones. To decipher which bone cells were possible targets of the irisin effect in ovx mice, we performed histological analysis to quantify the number of osteoclasts and osteoblasts (active or quiescent) per bone perimeter. In addition, we conducted an analysis of osteoblast differentiation from bone marrow harvested from ovx mice and cultured ex vivo, in parallel with gene expression analysis of the activating transcription factor 4 (*Atf4*), osteoprotegerin (*Opg*), and receptor activator of NF-kB ligand (*RankL*), key regulators for the differentiation and activity of these cells.

## 2. Results

### 2.1. Effect of Sham/Ovariectomy Surgery on Uterus and Tube Weight of Mice

**Figure 1:**
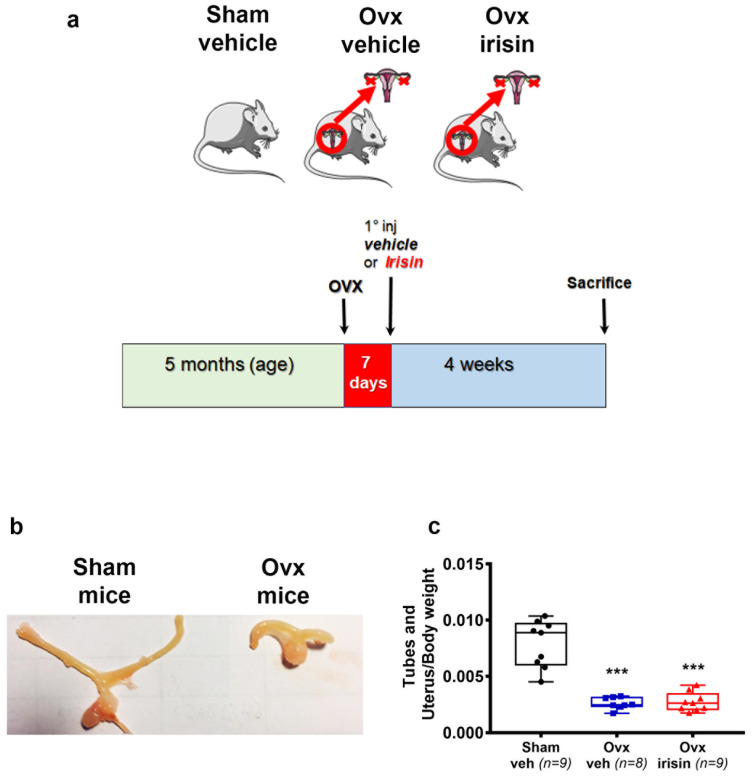
Exp. (**a**) Timeline of the study. (**b**) Photograph representative of tubes and uterus in sham-operated (Sham-veh) and ovariectomized (Ovx-veh) mice. (**c**) Changes in weight of tubes and uterus, normalized to body weight in Sham-veh (*n* = 9), Ovx-veh (*n* = 8) and Ovx-irisin (*n* = 9) mice. An ordinary one-way ANOVA with Tukey’s multiple comparisons tests was performed. Data are presented as a box-and-whisker plot with median and interquartile ranges, from max to min, with all data points shown. *** *p* < 0.001 vs. Sham-veh.

Our experimental design involved skeletally mature 5 month old mice subjected to bilateral ovariectomy; this was followed, after 7 days of recovery from surgery, by irisin treatment given intermittently (once a week) for 4 weeks (Figure 1a). To verify the success of the ovariectomy surgery, the wet weight of the uterus and tubes was evaluated at the time of the sacrifice. This analysis showed a significant reduction in uterus and tube weight in ovariectomized mice compared to sham mice (Ovx-veh: −67%, Ovx-irisin −65%) (Figure 1b,c).

### 2.2. Treatment with Irisin Prevents Trabecular Bone Loss in the Tibiae of Ovx Mice and Improves Their Mechanical Properties

**Figure 2:**
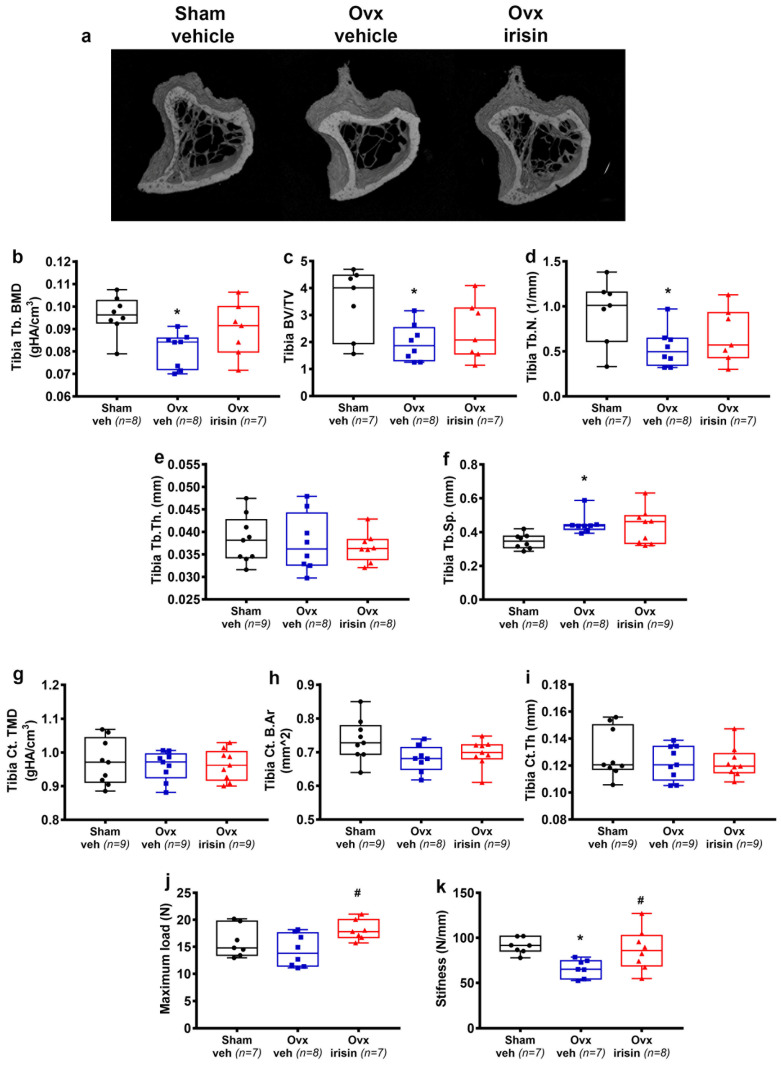
Treatment with irisin prevents trabecular bone loss in the tibiae of Ovx mice and improves their mechanical properties. (**a**) Representative micro-CT-generated section images and calculated trabecular and cortical parameters of tibiae harvested from Sham-veh mice, Ovx-veh mice and Ovx-irisin mice. (**b**–**f**) The trabecular bone parameters included bone mineral density (BMD) in Sham-veh (*n* = 8), Ovx-veh (*n* = 8) and Ovx-irisin (*n* = 7) mice; bone volume/total volume (BV/TV) in Sham-veh (*n* = 7), Ovx-veh (*n* = 8) and Ovx-irisin (*n* = 7) mice; trabecular number (Tb.N) in Sham-veh (*n* = 7), Ovx-veh (*n* = 8) and Ovx-irisin (*n* = 7) mice; trabecular thickness (Tb.Th) in Sham-veh (*n* = 9), Ovx-veh (*n* = 8) and Ovx-irisin (*n* = 8) mice; and trabecular separation (Tb Sp) in Sham-veh (*n* = 8), Ovx-veh (*n* = 8) and Ovx-irisin (*n* = 9) mice. (**g**–**i**) Cortical bone parameters included tissue mineral density (TMD) in Sham-veh (*n* = 9), Ovx-veh (*n* = 9) and Ovx-irisin (*n* = 9) mice; cortical bone area (Ct. B.Ar) in Sham-veh (*n* = 9), Ovx-veh (*n* = 8) and Ovx-irisin (*n* = 9) mice; and cortical thickness (Ct.Th) in Sham-veh (*n* = 9), Ovx-veh (*n* = 9) and Ovx-irisin (*n* = 9) mice. (**j**,**k**) The parameters of the biomechanical properties included maximum load in Sham-veh (*n* = 7), Ovx-veh (*n* = 8) and Ovx-irisin (*n* = 7) mice and stiffness in Sham-veh (*n* = 7), Ovx-veh (*n* = 7) and Ovx-irisin (*n* = 8) mice. A one-way ANOVA with Tukey’s multiple comparisons tests was performed in (**c**–**e**,**g**–**k**). Kruskal–Wallis with Dunn’s multiple comparison tests was performed in (**b**,**f**). Data are presented as box-and-whisker plots with median and interquartile ranges, from max to min, with all data points shown. * *p* < 0.05 vs. Sham-veh, # *p* < 0.05 vs. Ovx-veh.

To determine whether irisin protects against ovx-induced bone loss, we examined the long bones of Sham-veh, Ovx-veh, and Ovx-irisin mice. To assess changes in bone structure, we performed micro-CT analysis on the cortical and trabecular bones of the midshaft and proximal tibia, respectively. The results showed a significant reduction in BMD (−1.2 fold, *p* = 0.013), BV/TV (−1.7 fold, *p* = 0.03) and Tb.N. (−1.7 fold, *p* = 0.04) in the trabecular bone of the tibia in Ovx-veh mice compared to Sham-veh mice. In Ovx-irisin mice, however, the treatment prevented reductions in BMD, bone volume fraction (BV/TV), and Tb.N., which were unchanged compared to the control mice (Sham-veh) (Figure 2a,c,d). Likewise, in Ovx-veh mice, there was a significant increase in Tb.Sp. (1.3-fold, *p* = 0.02) compared to Sham-veh. However, in Ovx-irisin mice, Tb.Sp. was not significantly different from the control mice (Figure 2f). Differences were not found for Tb.Th. (Figure 2e) and for some parameters of cortical bone (TMD, B.Ar, and Ct.Th.) (Figure 2g,h,i). Furthermore, to determine whether irisin treatment influences the mechanical properties of the tibia, we evaluated maximum load and stiffness, which are the greatest load a bone structure withstands before fracturing and how much the entire bone deforms when loaded, respectively [32]. We observed that in Ovx-irisin mice, there was a significant increase in maximum load (1.3-fold, *p* = 0.04) (Figure 2j) and stiffness (1.3-fold, *p* = 0.04) compared to Ovx-veh. Noteworthy, the stiffness of the tibia was significantly reduced after ovariectomy (−34%, Ovx-veh vs. Sham-veh mice, *p* = 0.02) (Figure 2k).

### 2.3. Irisin Treatment Prevents Subchondral Bone Loss in the Tibiae of Ovx Mice

**Figure 3:**
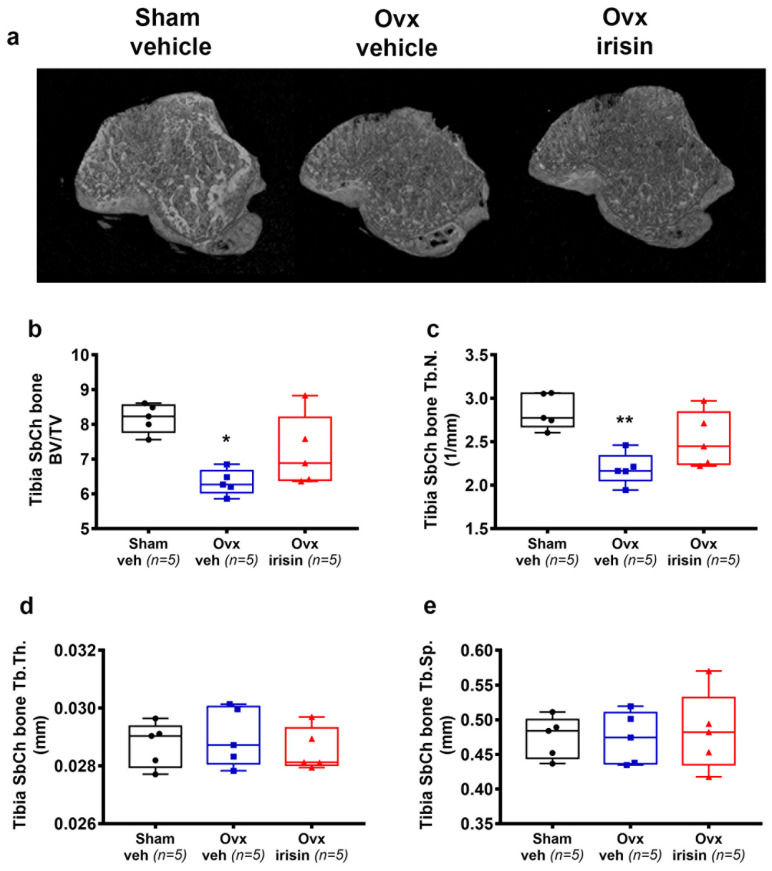
Irisin prevents subchondral bone loss in the tibiae of Ovx mice. (**a**) Representative micro-CT-generated section images and calculated parameters of subchondral bone in the tibiae harvested from Sham-veh mice, Ovx-veh mice and Ovx-irisin mice. (**b**–**e**) Trabecular bone parameters included bone volume/total volume (BV/TV), trabecular number (Tb.N), trabecular thickness (Tb.Th) and trabecular separation (Tb Sp) in Sham-veh (*n* = 5), Ovx-veh (*n* = 5) and Ovx-irisin (*n* = 5) mice. Kruskal–Wallis with Dunn’s multiple comparison tests was performed. Data are presented as box-and-whisker plots with median and interquartile ranges, from max to min, with all data points shown. * *p* < 0.05, ** *p* < 0.01 vs. Sham-veh.

In Ovx-veh mice, there was a significant decrease in tibial subchondral BV/TV (−23%, *p* = 0.01) (Figure 3b) and Tb.N. (*p* = 0.007) (Figure 3c) compared to the Sham-veh mice, whereas Tb.Th. and Tb.Sp. were unchanged (Figure 3d,e). However, if ovariectomized mice were treated with irisin, no significant loss of BV/TV and Tb.N. was observed compared with sham-operated mice (Figure 3b,c).

### 2.4. Treatment with Irisin Prevents Trabecular Bone Loss in the Femur of Ovx Mice

**Figure 4:**
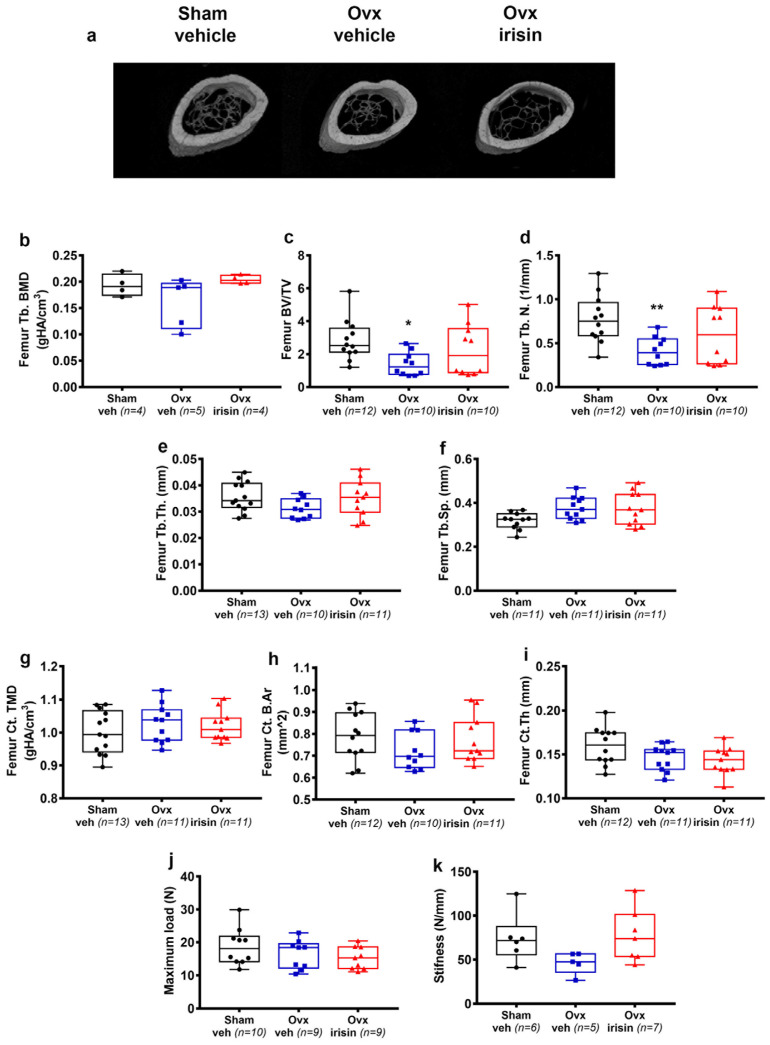
Treatment with irisin prevents trabecular bone loss in the femurs of Ovx mice. (**a**) Representative micro-CT-generated section images and calculated cortical and trabecular parameters of femurs harvested from Sham-veh mice, Ovx-veh mice and Ovx-irisin mice. (**b**–**f**) Trabecular bone parameters included bone mineral density (BMD) in Sham-veh (*n* = 4), Ovx-veh (*n* = 5) and Ovx-irisin (*n* = 4) mice; bone volume/total volume (BV/TV) in Sham-veh (*n* = 12), Ovx-veh (*n* = 10) and Ovx-irisin (*n* = 10) mice; trabecular number (Tb.N) in Sham-veh (*n* = 12), Ovx-veh (*n* = 10) and Ovx-irisin (*n* = 10) mice; trabecular thickness (Tb.Th) in Sham-veh (*n* = 13), Ovx-veh (*n* = 10) and Ovx-irisin (*n* = 11) mice; and trabecular separation (Tb Sp) in Sham-veh (*n* = 11), Ovx-veh (*n* = 11) and Ovx-irisin (*n* = 10) mice. (**g**–**i**) Cortical bone parameters included tissue mineral density (TMD) in Sham-veh (*n* = 13), Ovx-veh (*n* = 11) and Ovx-irisin (*n* = 11) mice; cortical bone area (Ct. B.Ar) in Sham-veh (*n* = 12), Ovx-veh (*n* = 10) and Ovx-irisin (*n* = 11) mice; and cortical thickness (Ct.Th) in Sham-veh (*n* = 12), Ovx-veh (*n* = 11) and Ovx-irisin (*n* = 11) mice. (**j**,**k**) The parameters of the biomechanical properties included maximum load in Sham-veh (*n* = 10), Ovx-veh (*n* = 9) and Ovx-irisin (*n* = 9) mice and stiffness in Sham-veh (*n* = 6), Ovx-veh (*n* = 5) and Ovx-irisin (*n* = 7) mice. A one-way ANOVA with Tukey’s multiple comparisons tests was performed in (**d**–**j**). Kruskal–Wallis with Dunn’s multiple comparison tests was performed in (**b**,**c**,**k**). Data are presented as box-and-whisker plots with median and interquartile ranges, from max to min, with all data points shown. * *p* < 0.05, ** *p* < 0.01 vs. Sham-veh.

Trabecular bone loss was also evident in the femurs, although to a lesser extent compared to the tibia. Through micro-CT analysis of femurs, we found that BV/TV (−51%, *p* = 0.02) (Figure 4c) and Tb.N. (−47%, *p* = 0.009) (Figure 4d) were significantly lower in femurs from Ovx-veh compared to Sham-veh. However, if ovx mice were treated with irisin, they showed no significant loss of BV/TV and Tb.N. compared with control mice (Sham-veh) (Figure 4c,d). Unlike the tibia, ovariectomy did not affect BMD, Tb.Sp. (Figure 4b,f) and bone mechanical strength in the femurs (Figure 4j,k). Similarly to the tibia, no significant changes in Tb.Th. (Figure 4e), TMD, B.Ar, and Ct.Th. (Figure 4g–i) were observed in the femurs.

### 2.5. Irisin Treatment Inhibits Bone Loss in the Spines of Ovx Mice

**Figure 5:**
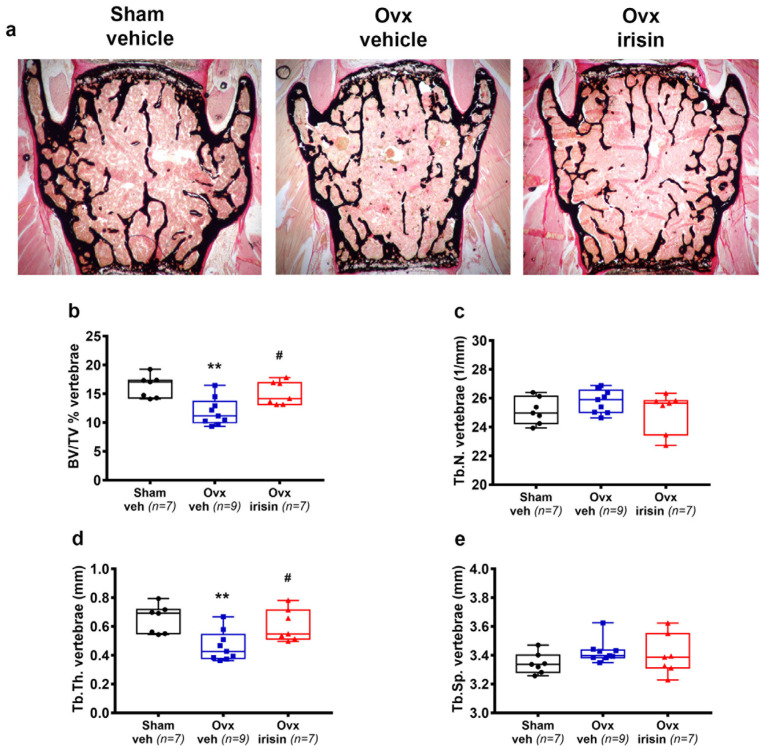
Irisin treatment inhibits bone loss in the spines of Ovx mice. (**a**) Von Kossa-stained vertebral sections and trabecular bone parameters from Sham-veh mice, Ovx-veh mice and Ovx-irisin mice (magnification 2×). (**b**–**e**) Trabecular bone parameters included bone volume/total volume (BV/TV) in Sham-veh (*n* = 7), Ovx-veh (*n* = 9) and Ovx-irisin (*n* = 7) mice; trabecular number (Tb.N) in Sham-veh (*n* = 7), Ovx-veh (*n* = 9) and Ovx-irisin (*n* = 7) mice; trabecular thickness (Tb.Th) in Sham-veh (*n* = 7), Ovx-veh (*n* = 9) and Ovx-irisin (*n* = 7) mice; and trabecular separation (Tb Sp) in Sham-veh (*n* = 7), Ovx-veh (*n* = 9) and Ovx-irisin (*n* = 7) mice. A one-way ANOVA with Tukey’s multiple comparisons tests was performed in (**b**–**d**). Kruskal–Wallis with Dunn’s multiple comparison tests was performed in (**e**). Data are presented as box-and-whisker plots with median and interquartile ranges, from max to min, with all data points shown. ** *p* < 0.01 vs. Sham-veh, # *p* < 0.05 vs. Ovx-veh.

We also investigated whether irisin was able to prevent ovariectomy-induced bone loss in vertebral bodies (Figure 5a). Through histological analysis of L3-L4 vertebra, we found that BV/TV in Ovx-veh mice was dramatically reduced compared to Sham-veh mice (−27%, *p* = 0.002), whereas Ovx-irisin mice displayed no loss of trabecular bone mass compared to Sham-veh mice, and significantly higher BV/TV than Ovx-veh mice (*p* = 0.021) (Figure 5b). The loss of bone mass in the vertebrae was mainly caused by a reduction in Tb.Th. (−29% Ovx-veh vs. Sham-veh, *p* = 0.005), whereas it was prevented by irisin treatment (*p* = 0.03 Ovx-irisin vs. Ovx-veh) (Figure 5d). No significant changes in Tb.N. and Tb.Sp. (Figure 5c,e) were observed.

### 2.6. Irisin Treatment Prevents Ovariectomy-Induced Bone Loss by Decreasing Osteoclast Number

**Figure 6:**
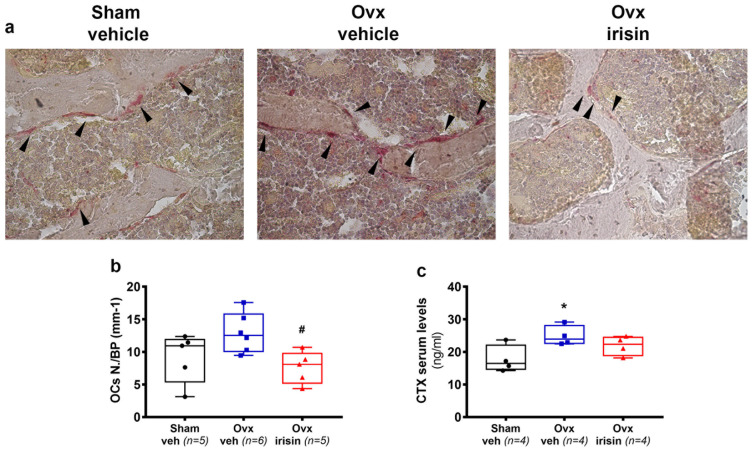
Treatment with irisin decreases osteoclast number in trabecular bone of Ovx mice. (**a**) Representative images of tartrate-resistant acid phosphatase-stained (Trap positive) osteoclasts (black arrowhead) in vertebral sections. (magnification: 40×). (**b**) Analysis of osteoclast number per bone perimeter (OCs N./BP) in Sham-veh (*n* = 5), Ovx-veh (*n* = 6) and Ovx-irisin (*n* = 5) mice. (**c**) Concentration of serum bone resorption marker CTX determined using an ELISA assay in Sham-veh (*n* = 4), Ovx-veh (*n* = 4) and Ovx-irisin (*n* = 4) mice. A one-way ANOVA with Tukey’s multiple comparisons tests was performed in (**c**). Kruskal–Wallis with Dunn’s multiple comparison tests was performed in (**b**). Data are presented as box-and-whisker plots with median and interquartile ranges, from max to min, with all data points shown. * *p* < 0.05 vs. Sham-veh, # *p* < 0.05 vs. Ovx-veh.

To decipher which bone cells were involved in irisin’s prevention of bone loss in ovx mice, we performed a histological analysis of trabecular bone in the vertebrae. As shown in Figure 6a, a higher number of trap-positive cells was observed in Ovx-veh mice compared to Sham-veh mice, thus proving that increased osteoclast formation was caused by ovariectomy. However, trap-positive cells were barely detectable in the trabecular bone of ovx-irisin mice. Using morphometric analysis, we quantified the OCs number per bone perimeter (OCsN./BPm), which showed a tendency to increase, albeit non-significantly, in Ovx-veh mice compared to Sham-veh mice; however, irisin treatment (Ovx-irisin) significantly reduced the number of OCs compared to Ovx-veh mice (−41%, *p* = 0.05) (Figure 6b). We sought to further characterize osteoclast activity by measuring the serum marker, collagen type 1 crosslinked C-telopeptide (CTX), which was significantly increased in Ovx-veh mice (+40%, *p* = 0.05) compared with Sham-veh mice (Figure 6c). However, if ovx mice were treated with irisin, they showed no significant change in CTX levels compared with control mice (Figure 6c).

### 2.7. Irisin Increases the Number of Osteoblasts in Trabecular Bone of Ovx Mice

**Figure 7:**
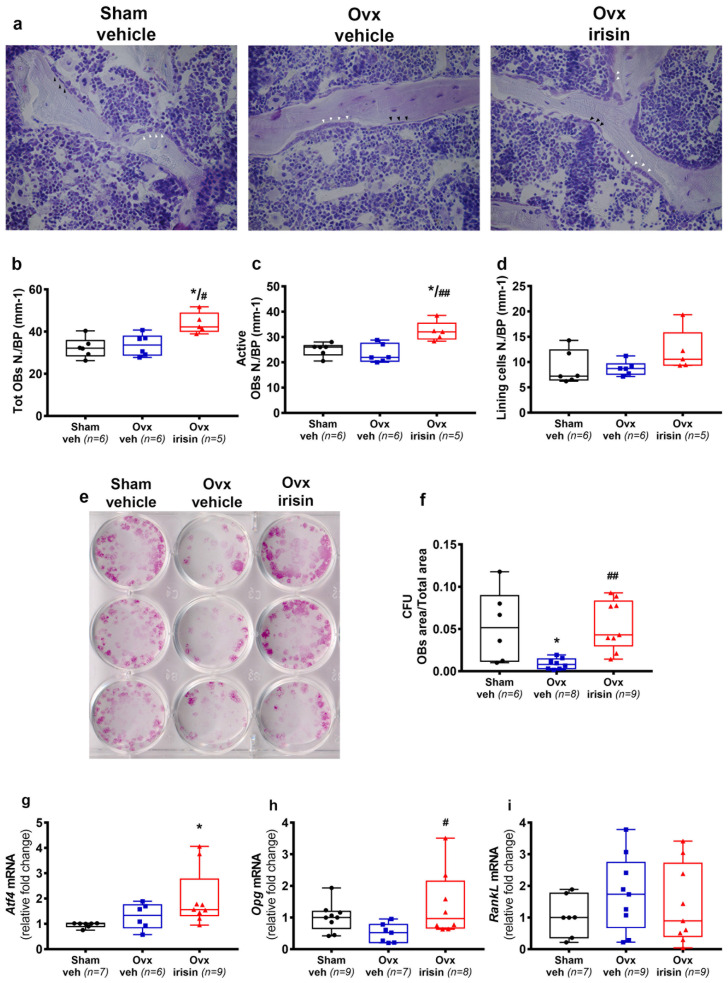
Figure **7.** Irisin treatment increases osteoblast number in Ovx mice. (**a**) Representative images of blue toluidine-stained osteoblasts in vertebral sections (magnification: 40×). (**b**–**d**) Analysis included osteoblast number per bone perimeter (OBs N./BP), active osteoblast number per bone perimeter (Active OBs N./BP) (white arrowhead) and bone lining cell number per bone perimeter (Lining cells N./BP) (black arrowhead) in Sham-veh (*n* = 6), Ovx-veh (*n* = 6) and Ovx-irisin (*n* = 5) mice. (**e**) Cfu-f formation in ex vivo cultures obtained from bone marrow harvested from Sham-veh (*n* = 6), Ovx-veh (*n* = 8) and Ovx-irisin (*n* = 9). (**f**) Quantification of Cfu-f formation. Quantification of mRNA expression for (**g**) *Atf4* in ex vivo culture of osteoblasts from Sham-veh (*n* = 7), Ovx-veh (*n* = 6) and Ovx-irisin (*n* = 9) mice, (**h**) *Opg* in ex vivo culture of osteoblasts from Sham-veh (*n* = 9), Ovx-veh (*n* = 7) and Ovx-irisin (*n* = 8) mice, and (**i**) *RankL* mRNA in ex vivo culture of osteoblasts from Sham-veh (*n* = 7), Ovx-veh (*n* = 9) and Ovx-irisin (*n* = 9) mice. A one-way ANOVA with Tukey’s multiple comparisons tests was performed in (**b**–**d**,**f**). Kruskal–Wallis with Dunn’s multiple comparison tests was performed in (**g**–**i**). Data are presented as box-and-whisker plots with median and interquartile ranges, from max to min, with all data points shown. * *p* < 0.05 vs. Sham-veh, # *p* < 0.05 or ## *p* < 0.01 vs. Ovx-veh.

Through histological analysis of the vertebrae, we quantified toluidine blue-stained osteoblasts (Figure 7a). Although the number of osteoblasts and bone lining cells (Figure 7b–d) was not affected by ovariectomy (not significant Ovx-veh vs. Sham-veh), we observed a significant increase in the total osteoblast number (OBs N./BP) and the number of active osteoblasts per bone perimeter (Active OBs N./BP) in Ovx-irisin mice compared with Ovx-veh mice (*p* = 0.04 and *p* = 0.01, respectively). Notably, independent of ovariectomy, irisin treatment increased the number of osteoblasts compared to Sham-veh mice (*p* = 0.02 and *p* = 0.04, respectively) (Figure 7b,c). To investigate more specifically the effect of irisin on osteoblastogenesis, and whether this was dependent on ovariectomy, we performed ex vivo culture of bone marrow mesenchymal cells harvested from Sham- and Ovx-mice (veh- or irisin-treated) and cultured with osteogenic medium for 10 days. In contrast to what we observed in vivo, cells harvested from Ovx-veh mice showed a significant reduction in osteoblast formation, measured as the area of colony-forming units (Cfu-f) at day 10, compared to Sham-veh (−84%, *p* = 0.04) (Figure 7f). Conversely, osteoblast formation in bone marrow cells harvested from Ovx-irisin mice was significantly higher than that in Ovx-veh mice (*p* = 0.003) (Figure 7f). In investigating the possible mechanism through which irisin increases osteoblast formation and activity in Ovx-mice, we investigated the pattern of gene expression in an ex vivo culture of osteoblasts, the main target cell of irisin. The results showed that, independent of ovariectomy, irisin stimulated the upregulation of mRNA for *Atf4* (Figure 7g), one of the key regulators of osteoblast differentiation [16]. Moreover, we found that the expression of *Opg* was enhanced by irisin (Figure 7h), without affecting *RankL* (Figure 7i), with the net effect of inhibiting of osteoclast formation.

### 2.8. Irisin Increases the Expression of Haptoglobin in Skeletal Muscle of Ovx Mice

**Figure 8:**
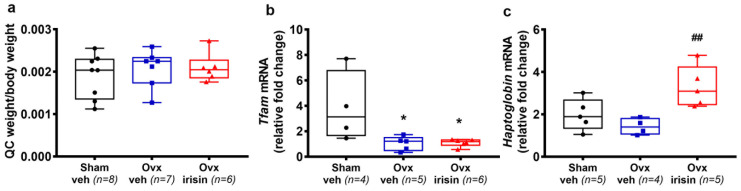
Figure **8.** Irisin treatment increases the expression of Haptoglobin in the skeletal muscle of Ovx mice. (**a**) Quadriceps weight normalized to total body weight in Sham-veh (*n* = 8), Ovx-veh (*n* = 7) and Ovx-irisin (*n* = 6) mice. Quantification of mRNA expression for (**b**) *Tfam* in quadriceps harvested from Sham-veh (*n* = 4), Ovx-veh (*n* = 5) and Ovx-irisin (*n* = 6) mice and (**c**) *Haptoglobin* in quadriceps harvested from Sham-veh (*n* = 5), Ovx-veh (*n* = 4) and Ovx-irisin (*n* = 5) mice. A one-way ANOVA with Tukey’s multiple comparisons tests was performed in a. Kruskal–Wallis with Dunn’s multiple comparison tests was performed in (**b**,**c**). Data are presented as box-and-whisker plots with median and interquartile ranges, from max to min, with all data points shown. * *p* < 0.05 vs. Sham-veh, ## *p* < 0.01 vs. Ovx-veh.

Since estradiol and irisin both play an important role in skeletal muscle regulation, we analyzed the quadriceps muscle of mice. Although there was no difference in the weight of the quadriceps (Figure 8a), molecular analysis revealed that the expression of the transcription factor A, mitochondrial (*Tfam*) was affected by ovariectomy (Figure 8b), thus suggesting an impairment of mitochondrial biogenesis caused by estrogen deficiency. Of note, in the quadriceps of Ovx irisin-treated mice, we detected a significant increase in *Haptoglobin* (Figure 8c), a key molecule modulated by irisin to prevent oxidative stress and muscle atrophy [33].

## 3. Discussion

The data obtained herein showed that irisin treatment in Ovx mice prevents trabecular bone loss in the long bones and spine by increasing the number of active osteoblasts, while decreasing osteoclasts. Interestingly, in the tibia, irisin also inhibits decreases in trabecular BMD and biomechanical properties, as well as BV/TV reduction in the subchondral bone.

The Ovx model is well accepted as an analog for simulating the effects of estrogen deficiency on bone [34]. Our experimental design involved skeletally mature 5 month old mice subjected to bilateral ovariectomy [34]; this was followed, after 7 days of recovery from surgery, by irisin treatment given intermittently (once a week) for 4 weeks. This treatment protocol has been shown to be effective in inducing bone anabolism in other mouse models of osteoporosis [35].

Since its discovery in 2012, experimental evidence indicates that the myokine irisin plays a role in the homeostasis of multiple organs and tissues [36,37]. Various studies have shown that the effects of this myokine are mainly related to the well-known benefits of exercise leading to stronger bones, increased energy expenditure, and improved cognitive abilities [10,17,38,39,40,41]. In this study, we found that irisin treatment prevents reductions in the trabecular BMD of the tibia, and prevents decreases in the BV/TV ratio of the tibiae, femurs and spines of ovx mice. In long bones, the irisin effect was mainly exerted through inhibiting reductions in Tb.N. In the spines, irisin prevented ovx-induced bone loss by preserving Tb.Th. at the levels of the control mice (sham-operated). At the cellular level, irisin inhibited the exacerbated ovx-induced osteoclast formation, and contributed to maintaining bone mass by increasing the osteoblasts’ number and activity.

**Figure A1:**
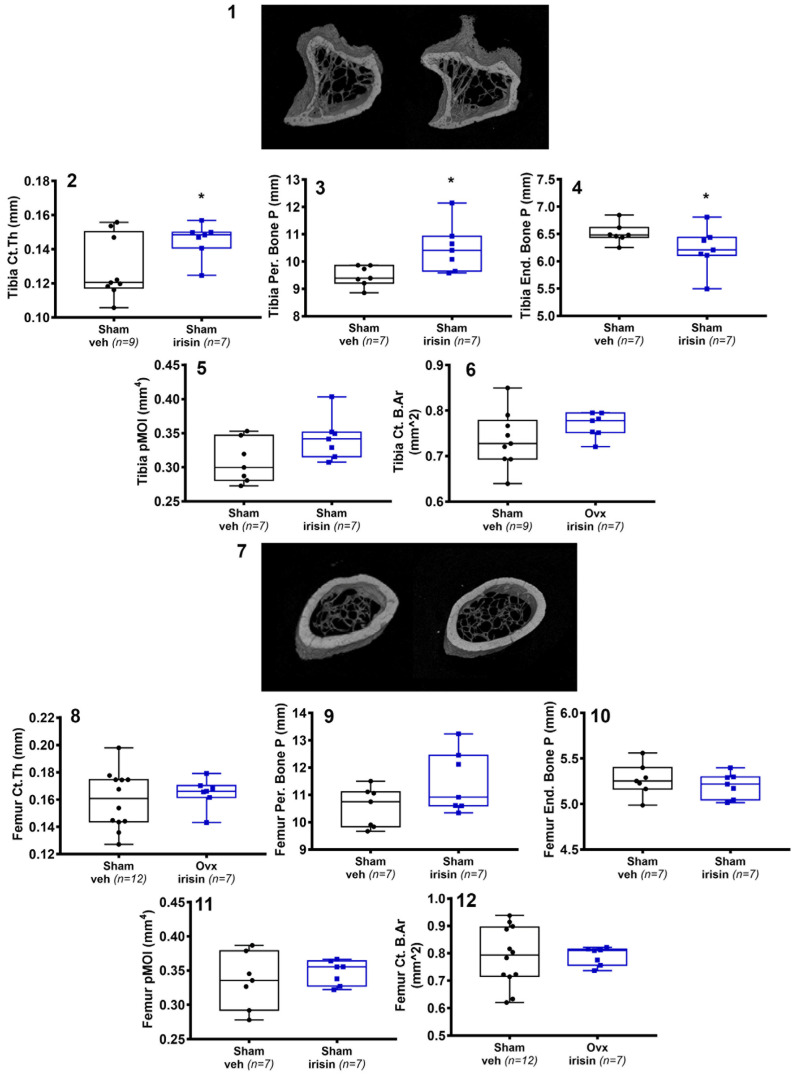
Representative micro-CT-generated section images of tibia harvested from Sham-veh mice and Sham-irisin mice (**1**). Cortical bone parameters included cortical thickness (Ct.Th) in Sham-veh (*n* = 9) and Sham-irisin (*n* = 7) mice (**2**), periosteal bone perimeter in Sham-veh (*n* = 7) and Sham-irisin (*n* = 7) mice (**3**), endosteal bone perimeter in Sham-veh (*n* = 7) and Sham-irisin (*n* = 7) mice (**4**), polar moment of inertia (pMOI) in Sham-veh (*n* = 7) and Sham-irisin (*n* = 7) mice (**5**), and cortical bone area (Ct. B.Ar) in Sham-veh (*n* = 9) and Sham-irisin (*n* = 7) mice (**6**). Representative micro-CT-generated section images of femurs harvested from Sham-veh mice and Sham-irisin mice (**7**). Cortical bone parameters included cortical thickness (Ct.Th) in Sham-veh (*n* = 12) and Sham-irisin (*n* = 7) mice (**8**), periosteal bone perimeter in Sham-veh (*n* = 7) and Sham-irisin (*n* = 7) mice (**9**), endosteal bone perimeter in Sham-veh (*n* = 7) and Sham-irisin (*n* = 7) mice (**10**), polar moment of inertia (pMOI) in Sham-veh (*n* = 7) and Sham-irisin (*n* = 7) mice (**11**), and cortical bone area (Ct. B.Ar) in Sham-veh (*n* = 12) and Sham-irisin (*n* = 7) mice (**12**). We performed an unpaired *t* test with Welch’s correction for values that passed the normality test (2, 6, 8, and 12), whereas for non-normal distributed values, we performed a Mann–Whitney test (3, 4, 5, 9, 19, and 11). Data are presented as box-and-whisker plots with median and interquartile ranges, from max to min, with all data points shown. * *p* < 0.05 vs. Sham-veh.

In postmenopausal women, loss of bone mass primarily affects trabecular bone, which declines in the first 5 years after menopause, and then continues to decline more slowly over the next 5 years. In contrast, cortical bone mass undergoes a significant loss 6–10 years after menopause, and becomes more severe 15 years after menopause [42]. Similarly, the effect on bone loss caused by ovariectomy was particularly evident at trabecular bone sites, even independently of the genetic background of mice, in both the vertebral body and proximal tibia. In comparison, cortical bone changes after OVX were generally less evident in mouse models of different strains [43]. Consistently, our results showed that estrogen withdrawal affects the trabecular compartment, but can be prevented by the action of irisin. We previously reported that in healthy male mice, irisin did not impact the trabecular bone mass [16]. Consistently, as shown in Appendix A, we found a significant increase in cortical thickness and the periosteal perimeter in the tibiae of Sham-irisin treated mice (internal control group) compared with Sham-veh mice (Figure A1), while no effect was detectable in trabecular bone. However, the effect of irisin on trabecular bone observed in ovx mice is in line with what we observed in the mouse model of disuse osteoporosis, wherein the trabecular compartment, particularly affected by unloading, was the primary target of irisin [18].

In OVX rodents, a decrease in the mechanical properties of bone, associated primarily with trabecular bone loss and then later with thinning of the cortex [44] has been observed. Likewise, in humans, the trabecular bone score (TBS) was introduced as a tool to quantify the three-dimensional microarchitecture of bone, and was more recently defined as an accurate marker of bone strength to predict skeletal fragility during the transition into menopause [45,46]. The maximum load, a measure of the greatest load that the bone structure can bear before fracturing, was thought to be solely dependent on cortical bone morphology. However, new evidence has highlighted that mechanical properties may also be predicted using architectural measures of trabecular bone [47,48]. In the present study, the three-point bending test showed that there was a significant decrease in tibial stiffness in Ovx mice, whereas irisin treatment prevented its reduction and increased the maximum load, suggesting that irisin played an important role in improving bone strength.

Some studies have shown a link between estrogen decline, the onset of osteoarthritis (OA) [49], and subchondral bone loss [4]. Consistent with this, we found that ovariectomy decreased the BV/TV ratio and the number of trabeculae (Tb.N.) in subchondral bone, whose decline was instead prevented by irisin treatment. The role of subchondral bone in the onset and progression of osteoarthritis has remained unclear for a long time. The debate focused on understanding whether osteoarthritis begins in cartilage (which, once damaged, negatively impacts the subchondral bone), or whether alteration of the latter can initiate the progression of osteoarthritis. However, data from several studies suggest that morphological changes in subchondral bone precede those in articular cartilage [50]. Therefore, the effect of irisin in preventing subchondral bone impairment would further strengthen the results obtained in other studies showing that treatment with irisin delays the development of osteoarthritis by protecting inflamed chondrocytes from apoptosis, oxidative damage, and underproduction of extracellular matrix [49,51,52].

It is also important to mention that irisin acts by coupling the anabolic effect on skeletal muscle and bone in mice [17,18]. Furthermore, human studies have shown that this myokine may be a marker of muscle and bone status in pathological conditions such as Charcot–Marie–Tooth neuropathy [53], or in bedrest studies [54].

Consistent with what we have shown here, in androgen-deficient mice (orchidectomized, Orx), irisin treatment protected against decreased trabecular BMD but did not affect weight loss in the soleus and tibialis anterior muscles, caused by orchidectomy [55]. However, that study did not include a biomolecular analysis of muscles to assess any possible irisin-mediated impact on mitochondrogenesis or muscle atrophy [17,18]. Like androgens, estradiol plays an important role in the regulation of skeletal muscle strength. Loss of muscle strength has been measured in Ovx mice, corresponding to the same reduction in postmenopausal women [56]. This sarcopenic phenotype is caused by increased apoptosis and changes in the functioning of myosin’s heavy chains [57], which are cellular pathways modulated by irisin in favor of skeletal muscle health. Of note, as already demonstrated by other authors in a mouse model of denervation-induced atrophy [33], we showed here that haptoglobin, which promotes skeletal muscle hypertrophy, was significantly upregulated by irisin treatment in the ovx mouse model.

Overall, the results obtained in this study provide new elements for a possible irisin-based anti-osteoporotic therapy for preserving the trabecular bone microarchitecture in tibiae, femurs, and spines under estrogen deficiency. Further studies on the Ovx mouse model are required to reveal the possible coupled effect, mediated by irisin, on muscle and bone to prevent or delay the onset of estrogen deficiency-induced osteosarcopenia.

## 4. Materials and Methods

### 4.1. Animals and Experimental Design

All experimental procedures were performed in strict accordance with the European Law Implementation of Directive 2010/63/EU, and Experimental protocols were reviewed and approved by the Veterinary Department of the Italian Ministry of Health (Project: 12-2022-PR). The 5 month old mice C57BL6 virgin female (*n* = 42) (Charles River, Wilmington, MA, USA) were randomly assigned to four groups: sham mice injected with vehicle [Sham-veh] or, as internal control, with irisin [Sham-irisin], and two groups of ovariectomized mice (ovariectomized vehicle-injected [Ovx-veh] and ovariectomized irisin-injected [Ovx-irisin].

### 4.2. Surgical Procedure for Ovariectomy

Before the surgical procedure, the mice had body weights of 22 ± 0.6 g Sham-veh, 23 ± 0.1 g Sham-irisin, 21.7 ± 0.6 g Ovx-veh and 22.5 ± 0.6 g Ovx-irisin. The mice were anesthetized according to the UCSF guidelines for rodent anesthesia with ketamine (100 mg/kg) (BP736, Sigma-Aldrich, Merck, Darmstadt, Germany) and xylazine (10 mg/kg) (1720407, Sigma-Aldrich, Merck, Darmstadt, Germany); the operator also followed IACUC guidelines for aseptic survival surgery. A 5 mm incision was made in the mouse, and the skin was detached from the underlying muscle before the incision of the latter was made. The ovary was gently exposed through the incision with blunt forceps, grasping the fat pad surrounding it. A hemostatic device was placed at the border between the oviduct and the uterus, and a ligature was placed near the uterus. A cut was made just above the hemostatic ligature, and the ovary and oviducts were removed with sterile forceps. It was only after the removal procedure that the hemostatic device was removed. At this point, the internal organs were repositioned in the abdomen. The muscle layer and skin were closed with absorbable sutures. Mice that underwent surgery without removal of the ovaries (Sham) were used as control. Sham mice were subjected to skin and muscle incision, exposure of the ovaries and ovarian fat, followed by closing with absorbable sutures and surgical site disinfection [34]. Following the surgical procedure, the animals were housed individually and kept under close observation for approximately 2–4 h until they fully recovered from anesthesia. The mice received post-operative therapy via subcutaneous injection twice daily for 3 days with the antibiotic enrofloxacin (20 mg/kg) (Vetranal, 33699, Sigma-Aldrich, Merck, Darmstadt, Germany) and the analgesic meloxicam (5 mg/kg) (444800, Sigma-Aldrich, Merck, Darmstadt, Germany). Following the recovery period (approximately 24 h after surgery), the animals were housed, four to five animals per cage, in a temperature-controlled environment with a 12 h light/dark cycle and with access to water and a regular diet ad libitum (Harlan Teklad 2019; SDS Special Diets Services, Witham, UK).

### 4.3. Treatment with Recombinant Irisin

One week after surgery, ovariectomized mice were treated with the vehicle (physiologic solution sterilized by 0.22 μ filtration) (*n* = 11) or with 100 μg/kg rec-irisin (AG-40B-0128-C010, Adipogen International, San Diego, CA, USA) (*n* =11) via intraperitoneal injection (i.p.) once a week for 4 weeks. Irisin was solubilized in sterile distilled water, following manufacturer’s instructions, as the lyophilized irisin powder contains lyophilized sodium phosphate buffer. The dose and timing of treatment were chosen because this treatment protocol has been shown to be effective in inducing bone anabolism in other mouse models of osteoporosis [10,17,18]. The group of Sham-veh mice (*n* = 13) received the vehicle, and the group of Sham-irisin mice (*n* = 7) received 100 μg/kg rec-irisin via i.p. injection once a week for 4 weeks. Mice were weighed once a week.

### 4.4. Animal Sacrifice and Tissue Harvesting

At the end of the experimental protocol of treatment, mice were euthanized with CO_2_ and their tissues surgically excised. Uterus, ovary, and quadriceps (QC) muscle weighing was performed and recorded. The success of ovariectomy was confirmed by measurement of at least a 75% reduction in uterine weight in the OVX groups relative to the Sham groups at the time of euthanasia. The L3–L4 vertebrae, right femur, and tibia were dissected, fixed with 4% (*v/v*) paraformaldehyde (158127, Sigma-Aldrich, Merck, Dramstadt, Germany) for 18 h at 4 °C, and processed for histological analysis, microarchitecture analysis, and a three-point bending test. Bone segments of the left limb were used for ex vivo experiments. Experimental procedures were carried out following the standard biosecurity and institutional safety procedures. Investigators were blinded to the group allocation. Power analysis: for an of 0.05 and *p* < 0.05; *n* = 12 mice/group. Sample sizes were chosen based on pilot studies and prior related work.

### 4.5. Micro-CT Analysis

MicroCT (µCT) scanning was executed to measure the morphological indices of the metaphyseal regions of femurs and tibiae. Femurs and tibiae were stored in scanning medium 70% ethanol, inserted into the specimens following the vertical axis of the scanner, and rotated around their long axes; images were acquired using Bruker Skyscan 1172 version 1.5 (Bruker, Kontich, Belgium), with the following parameters: voxel size 6 µm^3^; peak tube potential = 59 kV; X-ray intensity = 167 µA; 0.5 mm aluminum foil; sample rotation = 360°; rotation step = 0.4°; frame averaging = 3; exposure time = 1185 ms. Raw images were reconstructed using SkyScan reconstruction software (NRecon version 1.6.10.1) (Bruker, Kontich, Belgium), thereby producing three-dimensional cross-sectional image data sets using a three-dimensional cone–beam algorithm. The following setup was used: unified attenuation (output) range = 0.01–0.15; data were corrected for possible misalignments of overlapping sub-scans; mild beam-hardening correction = 40%; and ring artifact correction = 5. The images obtained and used in the next step were stored in 8-bit PNG format. A set of three hydroxyapatite (HA) phantoms (0.25 and 0.75 g·cm^−3^, and 2 mm diameter) was scanned with the same setup, and used for calibration and to compute volumetric BMD and TMD. Structural indices were calculated on reconstructed images using Skyscan CT Analyzer (CTAn version 1.20.8.0) software (Bruker, Kontich, Belgium). To delineate the region of interest (ROI) for trabecular and subchondral bone, an irregular anatomical region of interest adjacent to the endocortical boundary was chosen, drawn with an automatic algorithm. For cortical bone, a region of interest of regular and uniform shape was used. Cortical and trabecular bones were separated using a custom processing algorithm in CTAn, based on the different thicknesses of the structures. For femur analysis, cortical bone was assessed at the femoral midshaft. The scan volume of the femoral midshaft is centered on the midpoint of the bone, determined as half the distance from the distal condyle to the proximal point of the femoral head for all mice. Cortical bone was analyzed using a region of 150 slices, starting 9 mm distal to the metaphysis. Trabecular bone was analyzed in the proximal metaphysis region, starting proximal to the distal growth plate and continuing distally for 200 slices. For analyzing the properties of cortical bone, tibiae were scanned at the mid-diaphysis. starting 5.5 mm from proximal tibial condyles and extending for 200 6-µm slices (1.2 mm). For trabecular bone, tibiae were scanned starting 1.9 mm from the proximal tibial condyles, just distal to the growth plate, in the direction of the metaphysis, and extending for 200 slices (1.2 mm). The volume of interest (VOI) included the subchondral trabecular bone, starting below the subchondral plate and extending distally towards the growth plate, excluding both the cortical bone and growth plate interface. For tibiae subchondral bone, the volume of interest (VOI) consisted of a stack of ROIs drawn over 100 cross-sections, resulting in a height of 0.85 mm. The trabecular parameters included bone mineral density (BMD), bone volume fraction (BV/TV), trabecular number (Tb.N.), trabecular thickness (Tb.Th.), and trabecular separation (Tb.Sp.). The cortical parameters included tissue mineral density (TMD), cortical thickness (Ct.Th.), bone area (B.Ar), periosteal bone perimeter (Per Bone P), endosteal bone perimeter (End Bone P), polar moment of inertia (pMOI) (5) and cortical bone area (Ct. B.Ar). All methods of acquisition, image analyses, and terminology parameters analyzed were in line with the guidelines of Bouxsein et al. [58].

### 4.6. 3-Point Bending Test of Mouse Femurs and Tibiae

The tests were carried out with a Zwick tensile test machine (ZwickiLine Z1.0) (sn: 734188-2019, Zwick Roell, Ulm, Germany) with a 200 N load cell and the following test parameters: distance of lower supports: 8 mm; pre-load: 0.1 N; speed until pre-load: 20 mm/min. A loading rate of 1 mm/min was applied in the medial to the lateral direction. The software for controlling the machine and recording and analyzing data was Zwich/Roell testXpert III version 1.4. The bones were stored in 70% ethanol at 4 °C before testing and taken out to be rehydrated and stored at room temperature in good time before the mechanical test (>30 min). The length and diameter at the midshaft in the direction of the break force were measured for all the samples before testing. The samples were placed similarly on the supports, with the distal end to the right and the proximal side to the left. The posterior surface was facing down. The bones were freed from any soft tissue, and for the tibia, the fibula was removed from those samples in which it was still attached. Displacement (d) and force (F) were used to calculate parameters outlining whole bone structural properties. The parameters considered were stiffness (N/mm) and maximum load (N) [32,59].

### 4.7. Histological Analysis of Vertebrae

Lumbar vertebrae were embedded with methyl methacrylate (MMA) after dehydration, and the plastic sections were cut using a standard microtome (RM-2155 Leica, Heidelberg, Germany) into 7 μm for von Kossa staining, and 5 μm for tartrate-resistant acid phosphatase (TRAP) (387A, Sigma-Aldrich, Merck, Darmstadt, Germany) and blue toluidine staining (89640, Sigma-Aldrich, Merck, Darmstadt, Germany). The sections were stained using von Kossa silver impregnation (1.00362, Sigma-Aldrich, Merck, Darmstadt, Germany) with the van Gieson (1.15974, Sigma-Aldrich, Merck, Darmstadt, Germany) counterstained method, in order to determine the cancellous bone volume fraction (BV/TV %), trabecular number (Tb.N., 1/mm) trabecular thickness (Tb.Th., mm) and trabecular separation (Tb.Sp., mm) of L3-L4 vertebrae. For the analysis of osteoclasts (osteoclast number per bone perimeter, OCs/BP), bone sections were incubated in TRAP staining solution and then counterstained with methyl green (M8884, Sigma-Aldrich, Merck, Darmstadt, Germany). Blue toluidine staining was performed for the analysis of osteoblasts. Sections were evaluated under brightfield microscopy after blue toluidine staining to determine the osteoblast number (Obs N./BP), active osteoblast number (Active Obs N./BP), and bone lining cell number (lining cells N./BP). For the histomorphometric evaluation of the L3–L4 segments, two images were acquired at 2× (0.77 pixel/µm) magnification for both vertebral segments of each mouse. To analyze osteoclasts and osteoblasts, approximately nine to ten images per sample were acquired with a 40× objective. Histological sections were viewed under a microscope (Leica) using a 2× and 40× objective lens, and analyzed using Image-J software 1.53 t (NIH) [60,61] Osteoclasts were counted as TRAP-positive cells per bone perimeter (OCs N./BP mm^−1^). Active osteoblasts were counted as cuboidal cells lined up along the bone perimeter (Active OBs N./BP mm^−1^), showing active cytoplasm for protein synthesis, whereas lining cells were counted as flat (quiescent) cells lining the bone perimeter (Lining cell N./BP mm^−1^) [62].

### 4.8. Ex-Vivo Primary Cell Cultures

Bone marrow was obtained by flushing from mouse femurs and tibiae, and cultured in a density of 4 × 10^5^ cells/cm^2^ in 12-well plates in 500 µL of α-MEM (A1049001, gGibco, Thermo Fisher Scientific, Waltham, MA, USA), complemented with 10% (*v/v*) FBS (A5256801, Gibco, Thermo Fisher Scientific, Waltham, MA, USA) and 1% penicillin/streptomycin (15140122, Gibco, Thermo Fisher Scientific, Waltham, MA, USA). For osteogenic differentiation, bone marrow cells were cultured with α-MEM/10% FBS, supplemented with 5 μg/mL ascorbic acid (A4403, Sigma-Aldrich, Merck, Darmstadt, Germany) and 10^−2^ M β-glycerophosphate (G9422, Sigma-Aldrich, Merck, Darmstadt, Germany). At day 10, cells were fixed in 3.7% (*v/v*) formaldehyde for 5 min and subjected to alkaline phosphatase (ALP) staining (86R, Sigma-Aldrich, Merck, Darmstadt, Germany). The images were acquired with an appropriate scanner, and Image J software (version 1.53t) was used to calculate the area of the ALP+ colony-forming unit (Cfu-f) [63].

### 4.9. Real-Time PCR

Muscle biopsies were homogenized with Ultra-Turrax T8 (Ika, Staufen im Breisgau, Germany). Total RNA from muscle tissue and osteoblast ex vivo cultures was extracted using spin columns (Qiagen, Hilden, Germany). Reverse transcription was performed using iScript Reverse Transcription Supermix (Bio-Rad Laboratories, Hercules, CA, USA) in the thermocycler (My cycler; Bio-Rad Laboratories, Hercules, CA, USA). Real-time PCR on the CFX96 real-time system (Bio-Rad Laboratories, Hercules, CA, USA) was performed using SsoFast EvaGreen Supermix (Bio-Rad Laboratories, Hercules, CA, USA) for 40 cycles (denaturation 95 °C for 5 s; annealing/extension 60 °C for 10 s), after an initial 30 s long phase for enzyme activation at 95 °C. The primers used were designed with Primer Blast (https://www.ncbi.nlm.nih.gov/tools/primer-blast/, accessed on 14 May 2023). All primers span an exon–exon junction. The primer sequences were as follows: *Gapdh* (S-acaccagtagactccacgaca, AS-acggcaaattcaacggcacag); *Atf4* (S-gcctgactctgctgcttacattac, AS-cacgggaaccacctggagaag); *Opg* (S-gaccacctttatacggacag, AS-ctcacactcacacactcg); *RankL* (S-cccatcgggttcccataaagt, AS-cccgatgtttcatgatgccg); *Tfam* (S-taggcaccgtattgcgtgag; AS-cagacaagactgatagacgaggg); *Haptoglobin* (*Hp*) (S-gaatgtgaggcaggccaaga; AS-ccacgtagagcgttagggtg) and beta-2-Microglobulin (B2M) (S-tgctatccagaaaacccctca; AS-tttcaatgtgaggcgggtgg).

The geometric mean of two housekeeping genes (*GAPDH*, *B2M*) was used to normalize the data. Each transcript was analyzed in triplicate, and quantitative measures were calculated using the ΔΔCT method and expressed as a relative fold change from control [64].

### 4.10. ELISA Assay

Mice blood samples were collected at the sacrifice. Blood was collected into serum tubes and allowed to clot for 30 min at room temperature before centrifugation for 15 min at 1000× *g*. After centrifuging, the samples were aliquoted and stored at −80 °C until analysis. Sera were assayed for circulating levels of the C-terminal telopeptide of type I-collagen (CTX), using an ELISA kit (AC-02F1, Immunodiagnostic Systems, Boldon, UK) [65] and following the manufacturer’s protocol. Standard curves were generated using serial dilutions of the CTX calibration standards supplied in the ELISA kit.

### 4.11. Statistical Analysis

Analysis of sample distribution was performed using a D’Agostino and Pearson normality test. Parameters were expressed as the median and interquartile range (IQR) using GraphPad Prism 7 (GraphPad Software, Inc., La Jolla, CA, USA). For values that passed the normality test, we performed a one-way analysis of variance (ANOVA) with Tukey’s multiple comparisons tests, whereas for non-normal distributed values, we performed a Kruskal–Wallis test with two-group comparison by Dunn’s multiple comparisons tests. Every possible comparison was made among the groups: sham mice injected with vehicle [Sham-veh], ovariectomized mice (ovariectomized vehicle-injected) [Ovx-veh], and ovariectomized irisin-injected [Ovx-irisin]. All the comparisons were made by comparing the mean/rank of each group with the mean/rank of every other group. For microCT analysis, in Figure A1, which includes different parameters of cortical bone (periosteal bone perimeter, endosteal bone perimeter, polar moment of inertia and cortical bone area), comparisons were made between the internal control group [Sham-irisin] and the Sham-veh group, using an unpaired t test with Welch’s correction for values that passed the normality test; for non-normal distributed values, we performed a Mann–Whitney test. All data are presented as box-and-whisker plots with median and interquartile ranges, from max to min, with all data points shown. Differences were considered significant at *p* < 0.05.

## Data Availability

Data are available in a publicly accessible repository that does not issue DOIs. These data can be found here: https://1drv.ms/u/s!AuuLaz9ttim-goduVA8dV8T1ZsX_fQ?e=DIJow5, accessed on 23 May 2023.
